# Reproductive Diversity in Cultivated Tomato (*Solanum lycopersicum* L.): Relationships Among Floral, Fruit and Seed Traits

**DOI:** 10.3390/plants15060878

**Published:** 2026-03-12

**Authors:** Fabrizio Olivieri, Lorenzo Mancini, Barbara Farinon, Maurizio Enea Picarella, Andrea Mazzucato

**Affiliations:** Department of Agriculture and Forest Sciences (DAFNE), Tuscia University, Via S Camillo de Lellis Snc, 01100 Viterbo, Italy; fabrizio.olivieri@unitus.it (F.O.); lorenzo.mancini@unitus.it (L.M.); b.farinon@unitus.it (B.F.); picarella@unitus.it (M.E.P.)

**Keywords:** domestication, *Fasciated* gene, flower organ number, phenotyping, seed size, stigma position

## Abstract

The extensive diversification of flower shape and organs underpins the adaptive success of angiosperms. Despite substantial knowledge of the molecular mechanisms controlling flower induction and development, few studies have quantified the variability in floral traits within species or explored their correlation with other reproductive traits. In cultivated tomato (*Solanum lycopersicum* L.), human selection has driven fruit diversification in terms of size and shape. In the present study, 48 landraces representing tomato diversity in reproduction-related characteristics were phenotyped for 18 flower structural or dimensional traits. Flower traits exhibited lower coefficients of variation compared to other reproductive traits, though organ numbers showed high heritability values. Flower organ number and size were tightly correlated, but the correlation between dimensional traits was weaker. This likely reflects the selective pressures on pistil traits during domestication, including specific mutations affecting carpel number and ovary morphology. While ovary and fruit size were positively correlated, no relationship was found between ovule and seed size, suggesting that genes related to seed size generally act after fruit set. The collection was genotyped at the *Fasciated* (*Fas*) locus, and 13 floral traits were significantly different in *fas* mutants. The phenotypic variability described in this study could help breeders select for more fertile flowers and assist reproductive biologists in linking genes to flower development.

## 1. Introduction

Flowers underpin the extraordinary diversity of angiosperms, largely due to their highly efficient fertilization systems and their close coevolution with animal pollinators [1]. However, the adaptive success of angiosperms depends not only on the presence of flowers, but also on the remarkable diversification of their reproductive structures, which surpasses that of any other clade of land plants [2].

Throughout angiosperm history, transitions from outcrossing (allogamy) to self-fertilization (autogamy) have occurred repeatedly and represent one of the most common evolutionary trajectories among flowering plants [3]. In many cases, these transitions are associated with a characteristic suite of morphological and functional changes in floral traits, collectively referred to as the “selfing syndrome” [4,5]. Among these modifications, reductions in corolla size and alterations in the relative positions of the stamens and stigma are considered particularly important [3,6]. Other traits directly associated with the selfing syndrome include reductions in the pollen-to-ovule ratio, nectar production, and floral scent. The association between these floral traits and high selfing rates has been demonstrated across a broad taxonomic survey of angiosperms. For example, in the model species *Arabidopsis thaliana*, a high rate of self-pollination is correlated with reduced flower size compared to its outcrossing relative, *A. lyrata* [6].

The domestication of plant species by humans has often been accompanied by shifts in reproductive strategies [7]. These changes frequently involve an increase in self-fertilization, associated with reductions in floral size [8], floral longevity [9], and the distance between anthers and stigma. The rapid evolution of selfing-related traits has commonly been paralleled by the breakdown of self-incompatibility (SI) [10,11].

Evidence for selfing syndrome has likewise been reported in the tomato clade of the Solanaceae, particularly in the evolutionary history leading to tomato domestication [8]. The *Lycopersicon* section (*sensu* [12]) comprises the cultivated tomato (*Solanum lycopersicum* L.) and 12 wild relatives, all endemic to western South America. This section encompasses allogamous species with highly effective self-incompatibility systems and floral morphologies adapted for cross-pollination, as well as species exhibiting complete or partial self-compatibility and flowers adapted to self-pollination. The selfing syndrome in this clade therefore includes both the breakdown of self-incompatibility and the retraction of the stigma within the anther cone [13]. It is widely accepted that the closest wild relative of tomato is *S. pimpinellifolium* L., a self-compatible species that displays substantial variation in outcrossing rates and in the degree of stigma exsertion [14,15]. These traits are thought to correlate with a migration gradient from the center of origin, leading to increased rates of autogamy along this gradient [16]. Variation in stigma position persists in cultivated tomato; however, complete stigma exsertion is strongly reduced, thereby maximizing autogamy [16,17].

To date, comparative studies of tomato and its wild relatives have shown that variation in petal and sepal size represents the primary floral morphological traits associated with shifts in mating system [18]. Consistent with this general pattern, a transition from larger to smaller floral organs is observed between *S. chilense*, a member of the *Eriopersicon* group [12], and cultivated tomato [19]. Similarly, *S. pimpinellifolium* exhibits longer petals and shorter sepals compared with *S. lycopersicum* subsp. *cerasiforme* [13], indicating divergent evolutionary trajectories of perianth organs during domestication [20].

The identity of floral organs is established in the floral meristem through the activity of MADS-box transcription factors. According to the classical ABC combinatorial model, class A factors specify sepals in the first whorl; the combined activity of A and B factors specifies petals in the second whorl; B and C factors together specify stamens in the third whorl; and the C function alone specifies carpels in the innermost whorl [21]. Once specified, floral organs grow through an initial phase of cell proliferation, followed by a period of cell expansion. The final size and shape of mature floral organs therefore result from the combined effects of these two processes and can be altered by changes in the rate or duration of cell proliferation and/or cell expansion [22]. Numerous studies have shown that variation in floral organ size is typically governed by the combined action of multiple loci [23]. However, loci with large phenotypic effects can also play a significant role, driving substantial changes in organ size and contributing to the evolution of floral morphological novelties. Quantitative trait locus (QTL) analyses conducted in interspecific crosses between tomato and *S. pennellii* to investigate the genetic basis of natural variation in sepal and petal morphology identified tomato-derived QTL alleles that enhance both the size and number of these floral organs [24].

Despite the limited genetic variability of cultivated tomato—resulting from multiple bottlenecks during domestication and the development of modern cultivars [25]—variation at a relatively small number of genes affecting visual and organoleptic fruit traits has generated a remarkable diversity of fruit sizes, shapes, and colors [26,27]. Increases in fruit size (megalocarpy) have primarily been achieved through variation in genes regulating either cell number or cell size in ovary or developing fruit [28,29,30]. A major contribution to the evolution of larger tomato fruits also resulted from the selection of alleles affecting carpel number, notably mutations at the *FASCIATED* (*Fas*, [31]) and *LOCULE NUMBER* (*Lc*, [32]) loci. These mutations promoted the fixation of multilocular fruit phenotypes and the emergence of flattened and ribbed fruit forms. Such alleles are thought to have arisen prior to the introduction of tomato into Europe [33].

The *fas* variant consists of an inversion that alters the expression of two transcription factor genes, *SlYABBY2* and *SlCLAVATA3* (*SlCLV3*). Recent evidence indicates that the *fas* phenotype is primarily attributable to reduced *SlCLV3* activity [34,35]. The *lc* lesion is defined by two single-nucleotide polymorphisms (SNPs) located downstream of the *SlWUSCHEL* (*SlWUS*) stop codon [32]. Mutant alleles of *lc* and *fas* expand and maintain elevated *SlWUS* expression during carpel primordia development in the floral bud. This increase—resulting from a synergistic interaction between the two mutations—underlies the formation of multilocular fruits and produces a weak pleiotropic effect on the other floral whorls [36].

Variation in flower morphology within the tomato clade has largely been characterized by comparisons between wild and cultivated forms [18,20,24,37], whereas relatively few studies have explored the extent of variability within cultivated germplasm. In this study, we leveraged the extensive collection of cultivated tomato accessions assembled through the Traditom EU project [38,39] to analyze multiple floral traits in a subcollection of genotypes selected to maximize diversity in reproduction-related variables. This analysis revealed substantial variation in floral traits within cultivated tomatoes and provided insights into the evolutionary history of the species, major axes of phenotypic diversification, and trait correlations of potential relevance for future tomato genetics and breeding efforts.

## 2. Materials and Methods

### 2.1. Selection of Tomato Genotypes Diversified for Reproductive Traits

From the inventory of European tomato germplasm assembled within the Traditom project, a core collection (CC) designed to maximize the phenotypic and genetic diversity of salad tomatoes was selected. This CC included 226 tomato landrace accessions maintained in European countries, including France, Greece, Italy, and Spain, together with 39 commercial hybrids representing the major landrace fruit typologies [39]. Phenotyping of the Traditom CC was conducted in a field trial in Italy at the Experimental Farm of the University of Tuscia in Viterbo, Lazio (42°25′07″ N, 12°06′34″ E; 326 m a.s.l.), following the experimental design described by [39].

To identify a subset of accessions maximizing reproductive diversity within the CC, nine variables were selected and either retrieved or derived from previously published raw data [38,39]. Phenological traits included flowering date (FLOW; days from sowing until 50% of plants exhibited at least one open flower under uniform growing conditions) and ripening date (RIPE; days from sowing until 50% of plants produced at least one ripe fruit), both obtained from [39]. Floral morphology was characterized using stigma position [SP; score: 1, stigma inserted; 2, stigma at the level of the anther cone (flush stigma); 3, stigma slightly exserted (≤2 mm); and 4, stigma highly exserted (>2 mm)], retrieving data from [40].

Fruit-related traits included fruit set sequence (FSET; score: 1, very poor; 3, poor; 5, intermediate; 7, good; 9, very good); mean fruit weight on the first to fourth trusses (FW; g); fruit shape index (FRUSI; mean ratio of polar to equatorial diameter, measured on four fruits per plant); and soluble solid content (BRIX), measured on fruit juice using a digital pocket refractometer (ATAGO™ PAL-1, ATAGO Co., Ltd., Tokyo, Japan). These traits were collected as described [38,39]. Productivity traits included the total number of fruits per plant (NFR) and total estimated yield per plant (YIE; kg plant^−1^), calculated from NFR and FW, as reported in [39].

Two seed-related traits, not included in previous reports, were added to this set of variables. Seed weight (SW) was estimated by counting and weighing two replicates of 50 seeds per accession and expressed as mean unit seed weight (mg). The mean number of seeds per fruit (SxF) was subsequently estimated by weighing all seeds extracted from two fruit samples per accession (*n* ≥ 6) and dividing the total seed mass by SW.

During cultivation, the CC was sampled by collecting one open flower per plant (four per accession) from the second or third truss. In cases of disease symptoms or absence of flowers at the appropriate developmental stage, sampling was restricted to healthy plants bearing flowers at anthesis. Flowers were fixed in FAA solution (10% commercial formaldehyde, 50% ethanol, 5% acetic acid) for 2 days. Following fixation, flowers were transferred to 70% ethanol and stored at 4 °C until further observation.

To assemble a subcollection of accessions maximizing diversity across the 11 reproduction-related traits, an initial selection was performed by identifying accessions exhibiting extreme trait values. The selection was then refined, by including genotypes used as experimental controls in previous Traditom trials [38] and completed to 48 by manually choosing accessions to improve representation of all different tomato fruit types, all collections contributing to the CC, and to evenly sample the multivariate distribution obtained from the 11 traits.

### 2.2. Floral Trait Measurement

For each accession of the subcollection, four flowers were dissected using a razor blade to separate sepals, petals, stamens, and the pistil. The number of sepals (SEPN), petals (PETN), and stamens (STAN) were recorded. Micrographs of one representative organ from each of the first three floral whorls, as well as the pistil, were captured using a Canon PowerShot G6 digital camera (Canon, Tokyo, Japan). Image analysis was performed with ImageJ (Version is 1.53k) [41] to measure the length and width of sepals (SEPL, SEPW), petals (PETL, PETW), and stamens (STAL, STAW). For the pistil, style length and width (STYL, STYW) and ovary length and width (OVAL, OVAW) were measured. Total pistil length (PISL) was calculated as the sum of STYL and OVAL.

Each ovary was cross-sectioned under a stereomicroscope to determine the number of locules, corresponding to carpel number (CARN), and to isolate placental tissue containing ovules. Ovules were photographed using a stereomicroscope fitted with the digital camera, and images were used to estimate ovule longitudinal (OVUL) and transverse (OVUW) diameters. For each accession, four ovaries and at least 20 ovules per ovary were measured. Ovule measurements were averaged per ovary before statistical analysis. To parallel the estimation of FRUSI, the ovary shape index (OVASI) was calculated as OVAL/OVAW, and the ovule shape index (OVUSI) as OVUL/OVUW. Ovary size was estimated by calculating ovary area (OVAA). Considering the main ovary shapes (flat, round, and elongated), OVAA was approximated as the area of an ellipse using the formula: (½ OVAL × ½ OVAW × π). As ovules are always elliptic in shape, ovule area (OVUA) was calculated using the same approach. All metric variables were expressed in millimeters (mm) or square millimeters (mm^2^).

Pollen viability was assessed using four flowers per accession. From each flower, two stamens were individually dissected on a microscope slide, and pollen grains were released using a razor blade cutting longitudinally the whole pollen sac. Samples were stained with two drops of 1% (*w*/*v*) acetic orcein solution and examined under a light microscope (Axioskop 2 FS plus; Zeiss, Jena, Germany). For each slide, at least 100 pollen grains were counted and classified as stainable or non-stainable. Regular purple staining and morphological integrity were used as proxies for pollen viability (PV), as direct viability assays cannot be performed on fixed material. Pollen measurements were averaged per flower before statistical analysis and PV was finally expressed as the percentage of stainable pollen grains relative to the total number of pollen grains observed.

### 2.3. Genotyping of the Subcollection at the Fasciated Locus

Fresh leaves were collected from two representative, healthy plants per accession and ground in liquid nitrogen. Approximately 150–200 mg of frozen tissue was used for genomic DNA (gDNA) extraction following the protocol of [42]. Allelic variation at the *Fas* locus was assessed using three primers designed by [43]. Primers EP071 (5-CAGAAATCAGAGTCCAATTCCA-3) and EP1070 (5-ATGGTGGGGTTTTCTGTTCA-3) were used to detect the wild type *Fas* allele, while primers and EP1069 (5-CCAATGATAATTAAGATATTGTGACG-3) with EP1071 (acting as a reverse primer in the presence of the inversion) were used to amplify the mutant allele. These primers were combined in a multiplex PCR and functioned as a codominant marker system. PCR amplifications were performed in a final reaction volume of 10 µL, containing 1 µL of gDNA (20 ng/µL), 0.75 µL of each EP1070 and EP1071 primers (10 pmol/μL) and 0.5 µL of primer EP1069, 5 µL of 2× Green GoTaq buffer (Promega Corporation, Madison, WI, USA), and 2 µL of ddH_2_O. The PCR program consisted of an initial denaturation at 95 °C for 7 min, followed by 28 cycles of denaturation at 95 °C for 30 s, annealing at 59 °C for 30 s, and extension at 72 °C for 30 s, with a final elongation at 72 °C for 5 min. PCR products were resolved by electrophoresis on a 2% (*w*/*v*) agarose gels and visualized under UV illumination (312 nm) using a Gel Logic 100 imaging system (Kodak, Rochester, NY, USA) after staining with ethidium bromide.

### 2.4. Statistical Analysis

The initial dataset, comprising 190 genotypes and the 11 reproduction-related variables, was used to perform a principal component analysis (PCA) using the *factoextra* package in R (version 4.3.1). A second PCA was conducted on the 18 floral variables measured in the subset of 48 accessions selected to maximize variation in reproduction-related traits; results were graphically represented using *srplot* [44].

One-way analysis of variance (ANOVA) was performed using the General Linear Model procedure (PROC GLM) of the SAS software (SAS Institute Inc., Cary, NC, USA; https://www.sas.com/en_us/software/on-demand-for-academics.html, accessed on 20 February 2026). Homoscedasticity was evaluated using Bartlett’s test and, when necessary, corrected by appropriate variable transformation. For each trait, the coefficient of variation (CV) and broad-sense heritability (h^2^_ᴮ_) were estimated. Broad-sense heritability was calculated as h^2^_B_ = σ^2^_gen_/σ^2^_tot_, using variance components derived from one-way ANOVA. Genotypic variance (σ^2^_gen_) was estimated as (σ^2^_between_ − σ^2^_within_)/k, where *k* represents the number of replicates for observation. The within-group variance (σ^2^_within_) was considered an estimate of the environmental variance (σ^2^_e_), and total phenotypic variance (σ^2^_tot_) was calculated as the sum of σ^2^_gen_ and σ^2^_e_.

Pearson’s correlation coefficients among all measured traits (18 floral and 11 reproduction-related variables) were calculated using PROC CORR in SAS and visualized with *srplot*. Linear regression analyses were conducted using PROC REG in SAS, and the statistical significance of the models was assessed by one-way ANOVA.

## 3. Results

### 3.1. Selection of Tomato Accessions Diversified for Reproductive Traits

To construct a subcollection of genotypes maximizing diversity for reproduction-related traits, 16 accessions were initially selected based on extreme values for the traits considered (Supplementary Table S1). The selection was subsequently refined by adding 14 genotypes used as control accessions in the initial trials of the Traditom project [38]. The subcollection was completed with 18 additional accessions chosen to enhance representation of all major tomato fruit types, ensure contribution from all collections represented in the CC, and achieve homogeneous coverage of the PCA space derived from the 11 reproduction-related traits (Supplementary Figure S1). The final subcollection comprised 48 tomato landraces, including eight accessions from France, seven from Greece, two from Israel, 14 from Italy, and 17 from Spain (Supplementary Table S2). The selected accessions displayed high phenotypic diversity across the evaluated traits; for several variables, including FW, NFR, and SxF, variation spanned approximately two orders of magnitude (Supplementary Table S2).

### 3.2. Multivariate and Univariate Analysis of Floral Traits

**Figure 1:**
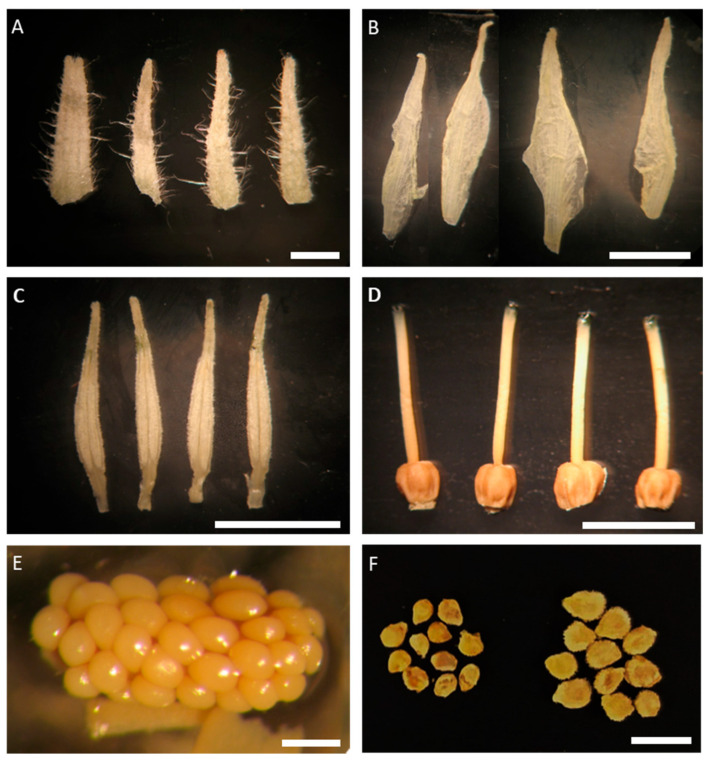
Representative pictures of floral organs dissected from FAA-fixed flowers and digitally analyzed: (**A**) sepals, (**B**) petals, (**C**) stamens, (**D**) pistils, (**E**) ovules, (**F**) seeds. Bars are 5 mm (**A**–**D**,**F**) and 0.2 mm (**E**).

**Figure 2:**
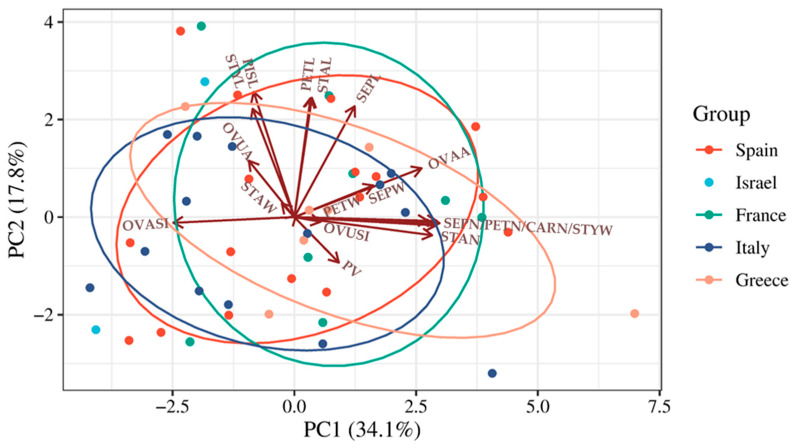
Distribution of the 48 studied accessions according to the first two principal components (PCs) and loading plot after multivariate analysis of 18 floral variables. Dots of different color indicate the country of provenience of the accession. Arrows represent original variables; their direction represents correlation between original variables and PCs; lengths represent devotion of original data to PCs. Traits considered were sepal (SEPN), petal (PETN), stamen (STAN) and carpel (CARN) number, sepal (SEPL), petal (PETL), stamen (STAL), style (STYL) and pistil (PISL) length, sepal (SEPW), petal (PETW), stamen (STAW), and style (STYW) width, ovary area (OVAA) and shape index (OVASI), ovule area (OVUA) and shape index (OVUSI), pollen viability (PV), date of flowering (FLOW) and ripening (RIPE), stigma position (SP), fruit set (FSET), fruit weight (FW), number of fruits per plant (NRF), estimated yield per plant (YIE), total soluble solids (°BRIX), seed weight (SW), number of seeds per fruit (SxF), and fruit shape index (FRUSI). Accessions belong to landrace collections from five countries, including Spain, Israel, France, Italy and Greece; ellipses represent 68% confidence interval for each origin group except Israel.

Flower dissection and morphometric analysis enabled the estimation of 18 floral trait variables (Figure 1). Multivariate analysis of these traits across the 48 accessions revealed that the first two principal components of the PCA accounted for 51.9% of the total phenotypic variation (Figure 2; Supplementary Table S3). The first principal component was mainly associated with traits related to floral organ number (SEPN, PETN, STAN, and CARN), including positively (STYW, OVAA) and negatively (OVASI) correlated traits. The second principal component was primarily driven by organ length-related variables, including SEPL, PETL, STAL, and STYL, as well as PISL (Figure 2; Supplementary Table S3). When accessions were grouped by country of origin, they were evenly distributed across the PCA space, indicating an absence of geographic structuring within the collection (Figure 2).

Five of the 18 floral variables, STAN, STYL, PISL, OVAA and PV, exhibited heterogeneous variances (*p* ≤ 0.01), but in all cases homoscedasticity was achieved through appropriate data transformation (Table 1). One-way ANOVA revealed significant differences among accessions for all floral traits, except for PETW and STAW (Table 1; Supplementary Table S2).

Several accessions repeatedly ranked among those with extreme trait values. Accession IS0010 displayed the lowest values for six traits, whereas TH2510 appeared five times among the highest-ranking values across the collection (Table 1).

**Figure 3:**
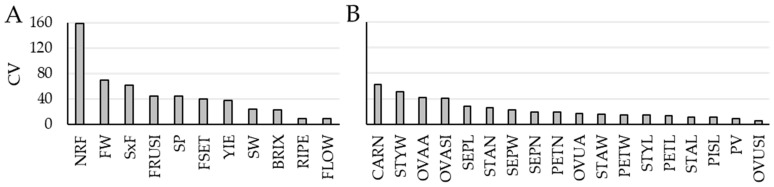
Values of the coefficient of variation (CV) calculated for (**A**) 11 reproduction-related and (**B**) 18 floral variables in 48 tomato accessions selected to maximize diversity in these traits. For variable abbreviation, see Materials and methods.

Variation in the 18 floral traits was compared with that of the 11 reproduction-related traits used for accession selection by means of the coefficient of variation (CV; Figure 3). The highest CV was observed for NFR. Overall, fruit-related reproductive traits exhibited higher CVs than floral variables. Among floral traits, four associated with pistil structure and size—CARN, STYW, OVAA, and OVASI—showed the greatest variability. In contrast, the lowest CVs were recorded for FLOW, RIPE, and OVUSI. SW displayed a CV approximately one-third that of FW (Figure 3; Supplementary Table S2).

Traits related to floral organ number exhibited high h^2^_B_, ranging from 0.56 to 0.80. Traits describing organ length showed intermediate h^2^_B_ values (0.35–0.71), whereas those related to organ width generally displayed the lowest estimates (0.10–0.33), with the notable exception of STYW (h^2^_B_ = 0.56). Very low heritability values were also observed for OVUSI and PV (Table 1).

### 3.3. Correlation Between Floral Organs and Other Reproductive Traits

**Figure 4:**
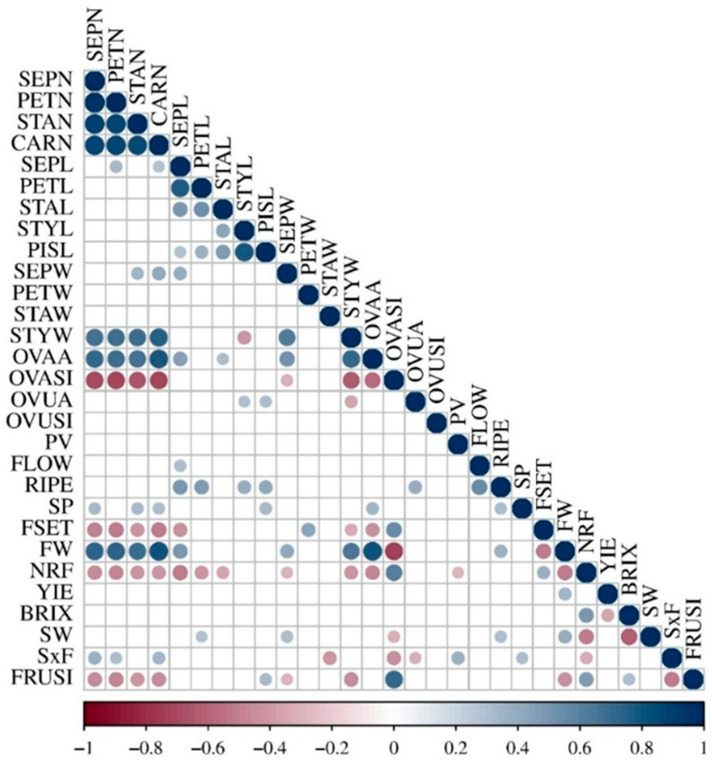
Pearson’s correlation coefficients among the 18 floral and the 11 reproduction-related traits. For variable abbreviation, see Materials and methods. Only significant correlations (*p* ≤ 0.05) are reported with a circle according to the color scale and proportional in size to the coefficient value.

Consistent with the PCA results, correlation analysis revealed strong intercorrelations among variables describing floral organ number, and to a lesser extent among traits related to organ length (Figure 4). Increases in floral organ number, which at the flower level were associated with wider styles and larger, flatter ovaries, were positively correlated at the fruit level with increased fruit size and higher seed number. Conversely, higher organ numbers were negatively correlated with FSET, NRF, and FRUSI. Notably, organ number traits were also positively correlated with SP.

FLOW and RIPE were strongly intercorrelated, as expected. However, RIPE showed additional strong positive correlations with sepal, petal, style, and total pistil length, and was also associated with increased OVUA (Figure 4).

The strong correlations among traits describing floral organ number were further confirmed by linear regression analyses. The most robust association was observed between SEPN and PETN, whereas the weakest relationship was detected between SEPN and CARN (Supplementary Figure S2A). Among length-related traits, SEPL and PETL showed a strong positive relationship, while associations between sepal length and lengths of organs from other floral whorls were weaker (Supplementary Figure S2B). In contrast, patterns for organ width differed markedly: regressions involving SEPW were not significant for PETW or STAW, but were highly significant for pistil-related traits, including STYW and OVAW (Supplementary Figure S2C).

**Figure 5:**
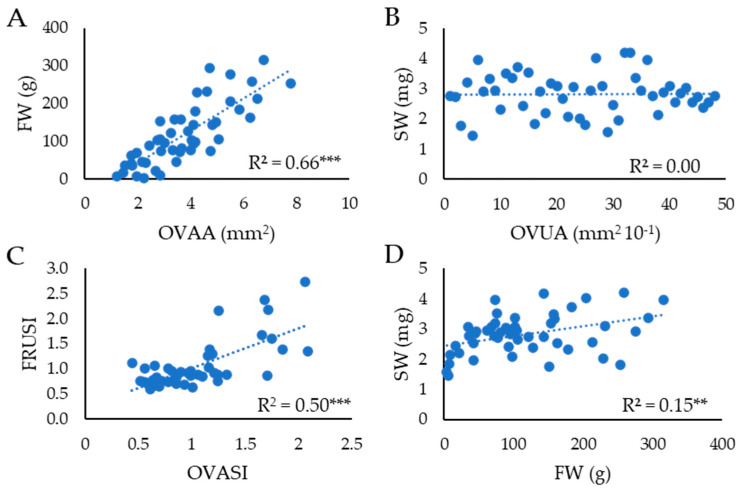
Linear regression analysis between (**A**) ovary area (OVAA) and fruit weight (FW), (**B**) ovule area (OVUA) and seed weight (SW), (**C**) ovary shape index (OVASI) and fruit shape index (FRUSI) and (**D**) fruit weight (FW) and seed weight (SW). The coefficient of determination of the regression (R^2^) is reported in each graph; ** and *** indicate regression significant for *p* ≤ 0.01 and 0.001, respectively.

Comparisons between floral traits and other reproduction-related variables revealed a strong positive correlation between OVAA and FW (R^2^ = 0.66, *p* ≤ 0.001; Figure 5A). In contrast, no significant relationship was detected between OVUA and SW (Figure 5B). A strong correlation was also observed between OVASI and FRUSI (R^2^ = 0.50, *p* ≤ 0.001; Figure 5C). Finally, FW and SW were weak but significantly correlated (R^2^ = 0.15, *p* ≤ 0.01; Figure 5D).

### 3.4. Effect of the Fas Mutation on Floral Organ and Reproductive Phenotypes

**Figure 6:**
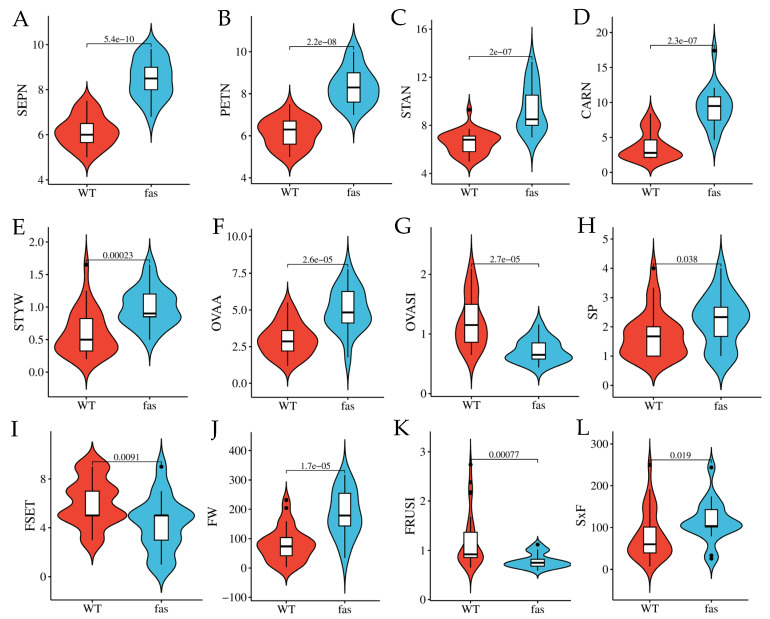
Floral trait means in tomato genotypes homozygous wild type (WT, red) and mutant (*fas,* light blue) for the *Fasciated* locus for floral and reproductive traits showing significant differences. Traits reported are number of sepals ((**A**), SEPN), petals ((**B**), PETN), stamens ((**C**), STAN) and carpels ((**D**), CARN), style width ((**E**), STYW), ovary area ((**F**), OVAA) and shape ((**G**), OVASI), stigma position ((**H**), SP), fruit set score ((**I**), FSET), fruit weight ((**J**), FW), fruit shape index ((**K**), FRUSI), and number of seeds per fruit ((**L**), SxF). *p* values for significance after Wilcoxon’s test are reported.

The collection was genotyped at the *Fas* locus using a multiplex marker targeting the inversion characteristic of the *fas* variant (Supplementary Figure S3). Among the 48 accessions, 32 were homozygous for the wild type allele (WT) and 16 were homozygous for the mutant *fas* allele (Supplementary Table S2). As expected, carpel number (CARN) differed significantly between the two genotype classes, with a mean value of 3.8 (range 2.0–8.4) for WT accessions and 9.6 (range 5.0–17.4) for *fas* mutants. Of the remaining 17 floral traits, 12 showed significant differences between WT and *fas* genotypes. These included all floral organ number traits (SEPN, PETN, and STAN), as well as traits related to pistil size (STYW, OVAA) and shape (OVASI; Figure 6A–G). In addition, *fas* accessions also exhibited higher SP (Figure 6H), reduced fruit set (Figure 6I), and produced larger and flatter fruits (Figure 6J,K) containing a higher number of seeds (Figure 6L).

## 4. Discussion

### 4.1. Floral Traits Showed High Diversity Within Cultivated Tomato

The evolutionary success of angiosperms is attributable not only to the presence of flowers, but also to the extraordinary diversification of their reproductive structures, which exceeds that observed in any other clade of land plants [1]. Floral morphological diversification has also been a major target of domestication, particularly in crop species where the edible product derives from reproductive organs.

Wild relatives of tomato typically bear flowers with five to six sepals, petals, and stamens, and produce bilocular fruits [45]. During domestication, cultivated tomato underwent pronounced modifications of floral architecture, largely driven by human selection for increased fruit size and diversification of fruit shape. As a result, some modern cultivars produce fruits that are up to two orders of magnitude larger than those of their wild progenitors [46]. This domestication-driven diversification has generated extensive floral and reproductive variability in cultivated tomato. In the present study, most of the traits analyzed exhibited substantial phenotypic variation across the 48 accessions selected to maximize reproductive diversity, with CVs ranging from 5.3% for OVUSI to 159.4% for NFR. At a broader scale, comparable extensive phenotypic diversity has been reported in the full Traditom collection, comprising 1499 accessions, where CV values for recorded traits ranged from 1% (fruit eccentricity and pericarp thickness) to 447% (distal-end fruit shape index) [38].

On average, floral traits displayed lower CVs than fruit-related traits, suggesting that floral morphology is more constrained by developmental and functional requirements, whereas fruit traits are more amenable to variation under human selection. Consistent with this interpretation, CVs exceeding 60% for FW have previously been reported in collections of cultivated tomatoes [38,47], as well as in spontaneous *S.l.* var. *cerasiforme* accessions [48].

Multivariate analysis indicated that most of the phenotypic variance was explained by traits related to floral organ number and organ length. Among these, the increase in CARN represents one of the pivotal events in tomato domestication, as human selection favored multilocular fruits because of their larger size [31]. Two major loci, *Lc* [32] and *Fas* [34], account for most of the variation in carpel number, giving rise to multilocular, large-fruited tomato types, such as flattened-ribbed, oxheart and pear-shaped cultivars. In cultivated tomatoes, the *lc* allele is more frequent than *fas* [33], and evidence suggests that these two loci act synergistically. While wild-type genotypes display a mean carpel number of 2.4, single *lc* and *fas* mutants exhibit mean values of 5.5 and 5.1, respectively, whereas double mutants reach an average of 10.5 carpels [33]. The PC1 axis captured both the direct and pleiotropic effects of the presence of the *fas* mutation. Indeed, all *fas* accessions (with one exception) were positioned in the positive quadrant of PC1 (Supplementary Figure S4).

The second principal axis of the multivariate analysis was primarily associated with traits describing floral organ length, which were strongly intercorrelated, suggesting that common genetic factors regulate the growth of these organs. Although sepal and petal length were positively correlated in the present study, previous work has shown that the evolutionary transition from allogamy to autogamy in tomato was accompanied by a general reduction in corolla size [18], while sepal size increased in cultivated forms [20]. Analysis of a panel of accessions spanning tomato domestication showed that the transition from *S. pimpinellifolium* to *S. lycopersicum* was associated with an increase in the number of sepals, petals, and carpels, whereas increases in organ size were observed only for sepals and carpels ([16]; Supplementary Figure S5).

Consistent with these findings, functional validation of the class C MADS-box gene *SlMBP21*, which encodes a negative regulator of cell expansion during sepal growth, demonstrated that knockout lines exhibit elongated sepals accompanied by improved fruit set [49]. If increased sepal length is associated with better fruit set, indirect selection for longer sepals during domestication could be hypothesized. Such an effect may be mediated by a protective role of sepals or by their contribution to photosynthetic activity, as hypothesized by [49] and is consistent with the reported selection of photosynthesis-related genes during tomato latitudinal migrations [15]. However, the mechanistic basis linking sepal size to fruit set has not yet been experimentally validated, and the molecular and physiological factors underlying this association remain to be elucidated. In contrast to these expectations, our screening revealed a negative relationship between fruit set and sepal length, with longer sepals tending to be associated with larger, multilocular fruits characterized by reduced fruit set.

With respect to organ width, SEPW was positively correlated with the size of pistil elements, including STYW and OVAA, traits that are in turn associated with increased carpel number and larger fruit and seed size. These correlations support the view that, after the pistil, the calyx represents the flower whorl most strongly affected by human selection, owing to its close association with carpel-related traits. Accordingly, sepal and carpel size may be regulated by shared genetic factors, resulting in pleiotropic effects like those exerted by the *ORGAN SIZE* (*ORG*) locus, which influences both floral and vegetative organ dimensions [50].

Although estimated in a single environment, the heritability values support the conclusion that the major genetic variation in tomato floral morphology is primarily driven by variants affecting organ numbers. Such traits showed positive correlations with organ length but not with organ width, except for STYW. Correlation between organ number and STYW was expected because style thickness is mainly due to the number of carpels composing the pistil. Among the floral whorls, sepals exhibited the highest genetic variation for size-related traits. In contrast, traits directly involved in reproductive function, such as ovule size, stamen width and pollen viability, displayed the lowest heritability estimates, indicating that these phenotypes are subjected to strong functional constraints and therefore tolerate limited variation. The low heritability of petal width could also be the effect of measurement noise, because petal tissue may fold and hamper a precise image analysis.

### 4.2. Floral Organ Number and Size Show High Correlation Among Different Floral Whorls

Multiple correlations were observed among the traits analyzed. While these relationships represent phenotypic associations rather than direct causal links, they offer important developmental insights and provide a basis for future functional investigations. As reported in previous studies, the number of elements in each floral whorl shows strong positive intercorrelation [24], suggesting the involvement of shared genetic determinants or of early-acting regulators operating at the onset of floral meristem differentiation. The *fas* variant represents a clear example of this phenomenon, as it causes an increased number of organs across all floral whorls. This pleiotropic effect is consistent with the very early expression of *CLV3* [36], at developmental stages that precedes the earliest phases of floral organogenesis. Notably, the first QTLs identified for SEPN and PETN were mapped to chromosomes 2 and 11, respectively, in genomic regions corresponding to the *Lc* and *Fas* loci [24].

Other transcription factors exhibit more spatially restricted phenotypic effects. The *excessive number of floral organs* (*eno*) mutant shows increased organ number in the three inner floral whorls, but not in sepals [51], indicating that its function likely acts downstream of, or later than, *Fas* during floral development. ENO regulates *SlWUS* expression to limit stem-cell proliferation in a flower-specific manner [52].

In cultivated tomato, a higher SP score has been positively associated with genotypes bearing flat, multilocular fruits [40]. Accordingly, we observed that on average SP values are significantly higher in *fas* genotypes compared with wild-type accessions. The mechanistic basis of this association remains unclear; elucidating its genetic and molecular underpinnings would be highly valuable for tomato breeding, as an exserted stigma can reduce self-pollination and consequently impair fruit set. Moreover, the association between fasciation severity and SP values was stronger under heat-stress conditions [40], suggesting that the development of *fas* cultivars adapted to high-temperature environments may pose greater breeding challenges.

### 4.3. Size and Shape of Ovary and Fruit Showed Significant Positive Correlation

Comparison of floral and mature fruit traits revealed, through regression analyses, strong positive correlations between ovary and fruit morphometric parameters, including both size and shape. These relationships indicate that a substantial proportion of fruit size and shape variability is already determined at the ovary stage, prior to anthesis. In addition to *Fas*, which influences both fruit size and shape, the major fruit size gene *FRUIT WEIGHT 2.2* (*fw2.2*), encoding a negative regulator of cell division, is expressed at very early stages of floral development [28]. Similarly, OVATE, a key determinant of fruit shape, is expressed during early flower development, and its effects are already detectable in ovaries at anthesis [53]. The mutation at the *Fas* locus is a key determinant of flat fruit morphology, and the ovary shape index (OVASI) effectively reproduced this phenotype, revealing significant differences between wild-type and *fas* mutant genotypes.

In contrast to ovary traits, ovule morphometric parameters, such as OVUA and OVUSI, exhibited limited variation across the analyzed genotypes and showed no correlation with SW. Although FW and SW were weakly correlated—consistent with previous reports [54]—mutations affecting ovary size did not appear to influence ovule morphology prior to anthesis. Ovule size at anthesis does not predict mature seed weight in this panel and the final seed mass is substantially determined by post-fertilization developmental processes. Because ovules play a highly constrained role in reproduction, their development is subject to strong functional limitations, resulting in reduced tolerance to phenotypic variation.

Despite this constraint, variation in seed weight is clearly present in tomato ([55]; this work), suggesting that genes underlying SW differences primarily act after pollination, during seed development rather than ovule formation. Although major fruit shape genes, such as *SUN*, *OVATE* and *fs8.1*, also influence seed size and shape [56], their effects are likely exerted during post-fertilization developmental stages, as no corresponding variation in ovule traits was detected in the germplasm analyzed here. Moderate variation in ovule dimensions has also been reported in barley [57]. However, in cereals—where genotypes have been directly selected for grain size—variation in ovule dimensions was correlated with caryopsis size [57], highlighting differences in selection pressures and developmental constraints between crop species.

## 5. Conclusions

The present analysis demonstrates that cultivated tomato, despite having experienced a domestication bottleneck and a concomitant reduction in genetic diversity, retains substantial variation in floral traits. This variability is closely associated with the diversity in fruit size and shape that emerged during the domestication process. The wide range of floral morphological variation detected in a well-diversified panel of European tomato landraces further indicates that reproductive traits are not structured by geographic origin. Several traits highlighted in this study warrant increased attention in future breeding programs. Manipulation of stigma position could be strategically exploited either to favor stigma insertion, thereby promoting autogamy and reproductive stability, or to enhance stigma exsertion to facilitate controlled crosses and hybrid seed production. Targeted genetic and breeding efforts may enable the uncoupling of *fas* genotypes from stigma exsertion, thus mitigating negative pleiotropic effects. The observed increase in sepal size—appearing as part of the tomato domestication syndrome alongside the more evident enlargement of pistil components—opens new avenues for investigating the pleiotropic and physiological changes accompanying human selection. Finally, elucidating the genetic determinants of seed size, together with their effects on seedling fitness and establishment, would provide valuable tools for breeding more resilient and vigorous tomato cultivars.

## Figures and Tables

**Table 1:** One-way ANOVA analysis on 18 variables describing floral traits in 48 selected tomato accessions, including homogeneity of variance (HOV) test, adopted data transformation, Fisher’s test significance, range of variable values, accessions showing the lowest and the highest value and broad sense heritability (h^2^_B_). For trait abbreviations, see Materials and methods.

Trait	HOV ^a^	Transformation	ANOVA ^a^	Range	Accession Showing Extreme Values:		h^2^_B_
					Lowest	Highest	
SEPN	ns		***	5.0–9.8	PO01510 VA0590	VI0870	0.65
PETN	*		***	6.5–21.0	BA1740 PO1510 VA0590	TH2510 VI0870	0.56
STAN	**	LOGx	***	5.0–13.3	IS0010	TH2510	0.60
CARN	ns		***	2.0–17.4	IS0010 PO1510 TH1470 VI0010 VI0060	TH2510	0.80
SEPL	ns		***	0.9–2.1	IS0010 TH0030 VA0590	VA2130	0.71
PETL	ns		***	9.8–18.5	IS0010	VA2290	0.43
STAL	ns		***	6.8–11.9	BA1740	MO1050	0.50
STYL	**	LOGx	***	3.6–9.4	PO1990	MO105	0.44
PISL	***	LOGx	***	7.3–11.9	TH2510	MO105	0.35
SEPW	ns		**	5.0–10.0	IS0010 MO0030	TH2510	0.33
PETW	ns		ns	2.0–4.0	VI0010	VI0060	0.12
STAW	ns		ns	1.0–1.7	IS0010 VA1300	VA0590	0.10
STYW	ns		***	0.2–1.7	BA1740 PO1510	PO1990	0.56
OVAA	**	LOGx	***	1.2–7.8	PO1510	TH2510	0.57
OVASI	ns		***	0.44–2.09	MO0040	PO2520	0.59
OVUA	*		***	0.014–0.030	MO0040	VA2290	0.57
OVUSI	ns		***	1.28–1.69	VA0590	VI0020	0.11
PV	***	arcsin	***	56.0–97.4	IS0030	TH0710	0.23

^a^ ns, not significant; *, **, ***, statistically significant for *p* ≤ 0.05, 0.01 and 0.001, respectively.

## Data Availability

All data represented in this study are available in the article and Supplementary Materials.

## Supplementary Materials

The following supporting information can be downloaded at: https://www.mdpi.com/article/10.3390/plants15060878/s1. Figure S1: Distribution of 190 Traditom Core Collection accessions according to the first (PC1) and second (PC2) axes after Principal Component Analysis on 11 reproduction-related traits; Figure S2: Regression analysis among floral traits in the 48 accessions of the studied collection; Figure S3: Examples of the genotyping carried out at the *Fasciated* (*Fas*) locus; Figure S4: Distribution of the 48 studied accessions according to the first two principal components (PCs) and loading plot after multivariate analysis of 18 floral variables. Figure S5: Analysis of variance of floral and reproduction-related data from [16] as distributed in *Solanum pimpinellifolium* (SPI), *S. lycopersicum* var. *cerasiforme* (SLC), and *S. lycopersicum* var. *lycopersicum* (SLL). Table S1: Traditom accessions phenotyped in the trial carried out in Italy and mean values for 11 reproduction-related traits; Table S2: Subcollection of 48 Traditom accessions maximizing phenotypic diversity in reproduction-related traits, mean values for 18 floral variables and genotype at the *Fasciated* locus; Table S3: Principal component analysis (PCA) carried out on the 48 accessions included in the subcollection with 18 floral trait variables.

## Abbreviations

The following abbreviations are used in this manuscript:

ANOVA	Analysis of variance
BRIX	Total soluble solids
CARN	Carpel number
CC	Core collection
*CLV3*	*CLAVATA3* gene
CV	Coefficient of variation
FAA	Formaldehyde, alcohol, acetic acid
Fas	*Fasciated* gene
FLOW	Flowering date
*Fs8.1*	*Fruit shape 8.1* gene/QTL
FSET	Fruit set sequence
FW	Fruit weight
*Fw2.2*	*Fruit weight 2.2* gene/QTL
GLM	General linear model
h^2^_B_	Broad sense heritability
Lc	Locule number gene
NFR	Number of fruits per plant
*O*	*Ovate* gene
*ORG*	*ORGAN SIZE* gene
OVAL	Ovary length
OVASI	Ovary shape index
OVAW	Ovary width
OVUL	Ovule length
OVUSI	Ovule shape index
OVUW	Ovule width
PCA	Principal component analysis
PETL	Petal length
PETN	Petal number
PETW	Petal width
PISL	Pistil length
PV	Pollen viability
RIPE	Ripening date
QTL	Quantitative trait locus
SxF	Number of seeds per fruit
SEPL	Sepal length
SEPN	Sepal number
SEPW	Sepal width
SNP	Single nucleotide polymorphism
STAL	Stamen length
STAN	Stamen number
STAW	Stamen width
STYL	Style length
STYW	Style width
SW	Seed weight
*WUS*	*Wuschel* gene
YIE	Estimated yield
