# Supplementary material for: Reproductive Diversity in Cultivated Tomato (Solanum lycopersicum L.): Relationships Among Floral, Fruit and Seed Traits
Source: Plants (Basel). 2026 Mar 12;15(6):878. doi: 10.3390/plants15060878 (PMC13029455; doi:10.3390/plants15060878)
Supplement: Supplementary file 1 [file plants-15-00878-s001.zip › Suppl figures.pdf]

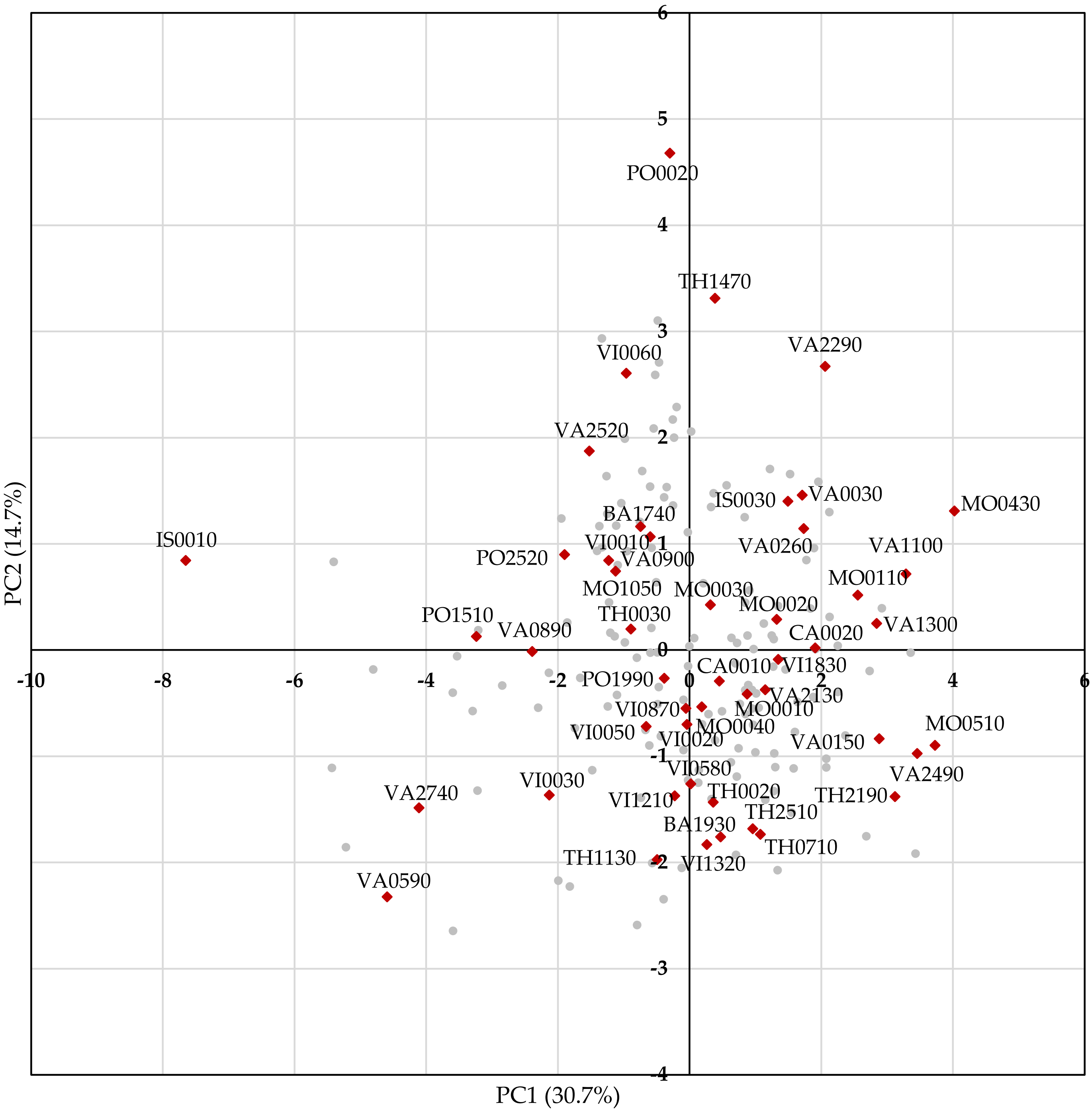

Supplementary Figure S1. Distribution of 190 Traditom Core Collection accessions according to the first (PC1) and second (PC2) axes after Principal Component Analysis on 11 reproduction-related traits. Codes are reported only for accessions selected in the sub collection maximizing reproduction-related traits (*red diamonds*).

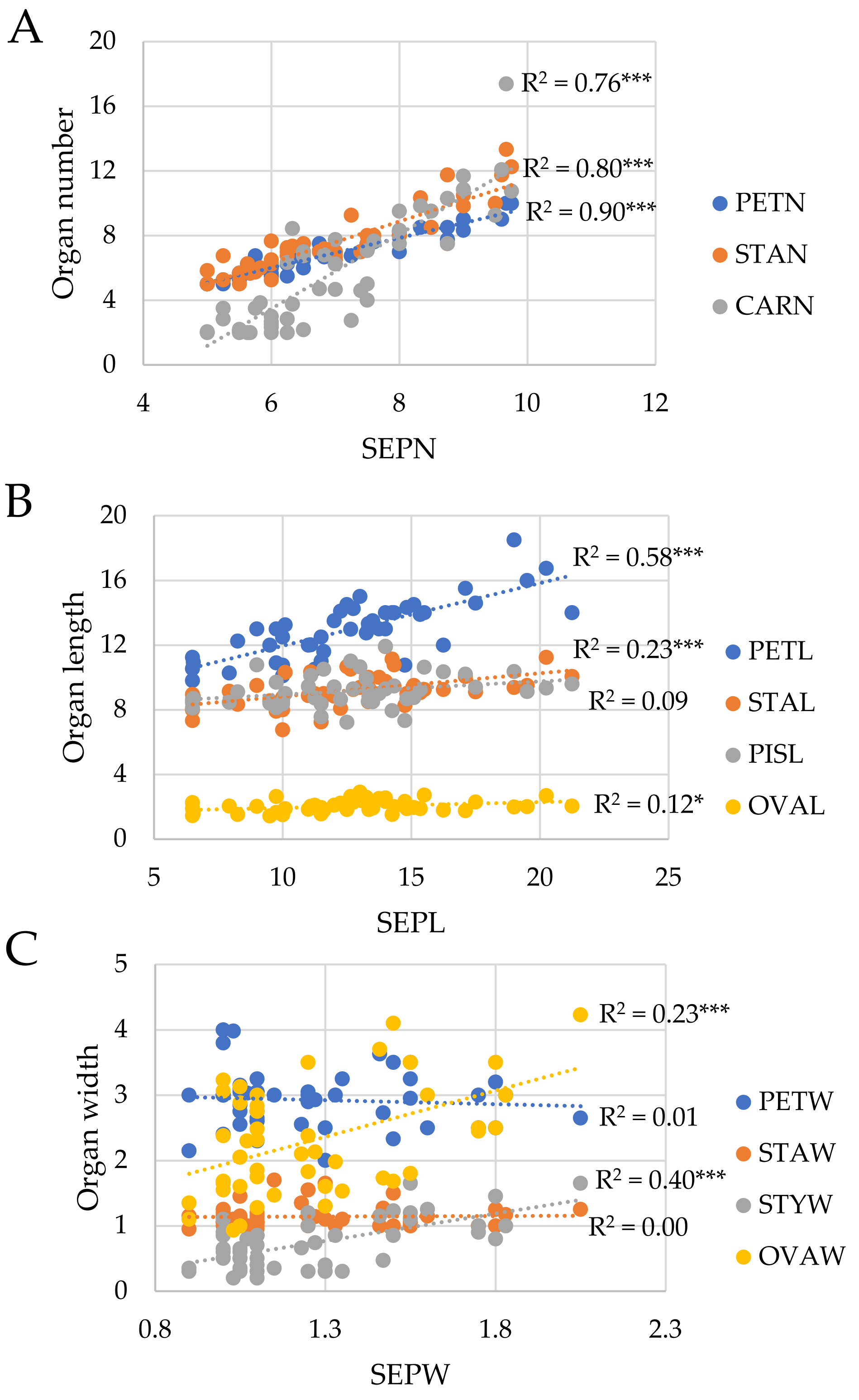

Supplementary Figure S2. Regression analysis among floral traits in the 48 accessions of the studied collection. (**A**) regression between the number of sepals (SEPN) and that of petals (PETN), stamens (STAN) and carpels (CARN); (**B**) regression between the length of sepal (SEPL) and that of petal (PETL), stamen (STAL), style (STYL) and ovary (OVAL); (**C**) regression between the width of sepal (SEPW) and that of petal (PETW), stamen (STAW), style (STYW) and ovary (OVAW). The coefficient of determination of the regression (R^2) is reported near each series; * and *** indicate regression significant for $p \leq 0.05$ and 0.001 respectively.

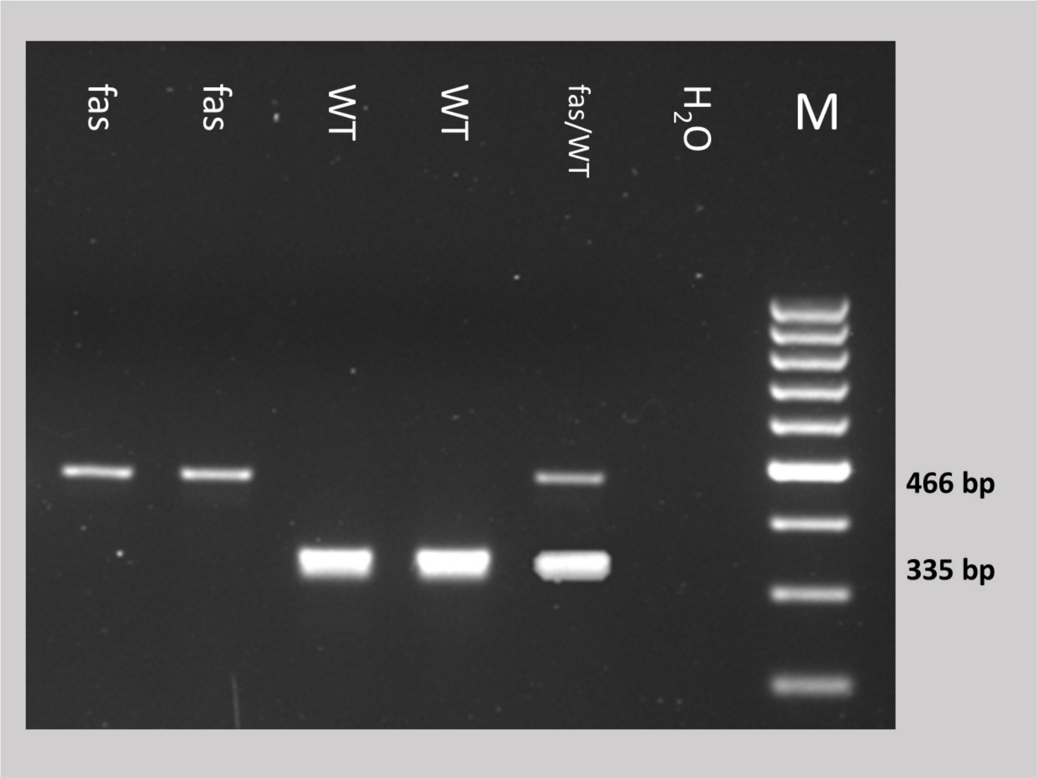

Supplementary Figure S3. Examples of the genotyping carried out at the *Fasciated* (*Fas*) locus. From left to right, PCR products of two plants homozygous for the *fas* mutation (fas), two plants homozygous for the wild type allele (WT), one heterozygote (fas/WT), a negative control and the molecular weight marker (M).

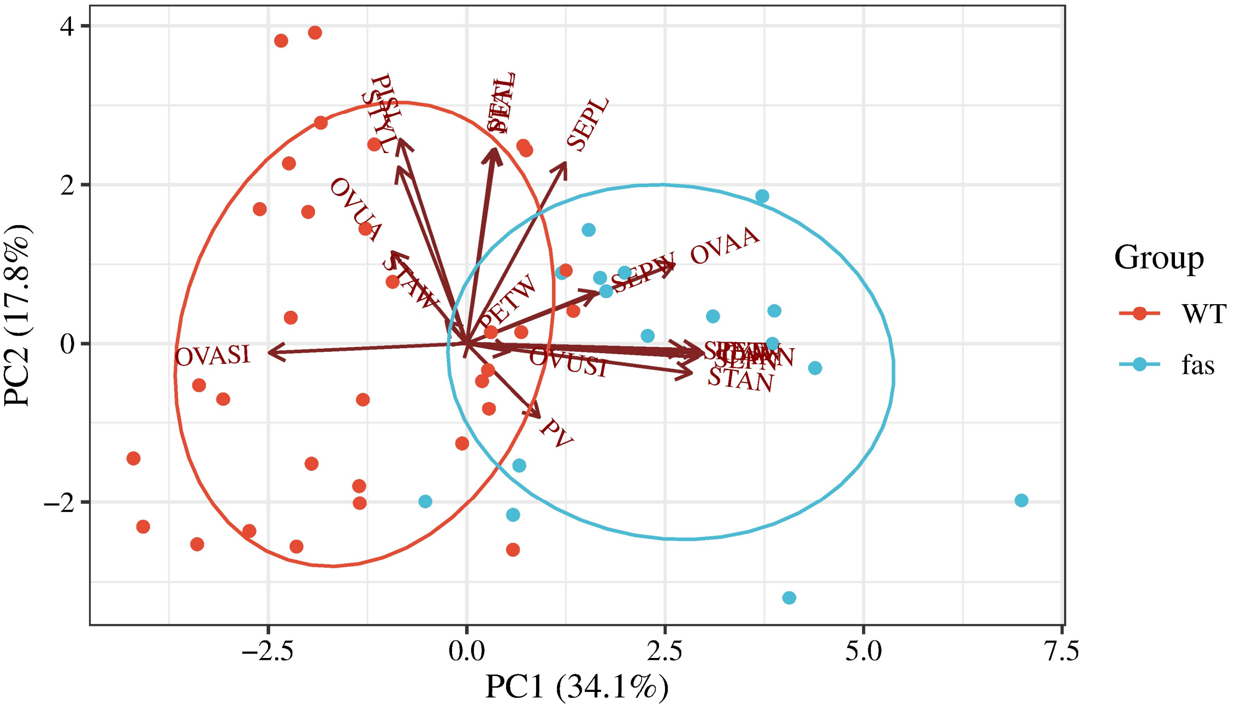

Supplementary Figure S4. Distribution of the 48 studied accessions according to the first two principal components (PCs) and loading plot after multivariate analysis of 18 floral variables. Arrows represent original variables; their direction represents correlation between original variables and PCs; lengths represent devotion of original data to PCs. Traits considered were sepal (SEPN), petal (PETN), sta-men (STAN) and carpel (CARN) number, sepal (SEPL), petal (PETL), stamen (STAL), style (STYL) and pistil (PISL) length, sepal (SEPW), petal (PETW), stamen (STAW), and style (STYW) width, ovary area (OVAA) and shape index (OVASI), ovule area (OVUA) and shape index (OVUSI), pollen viability (PV), date of flowering (FLOW) and ripening (RIPE), stigma position (SP), fruit set (FSET), fruit weight (FW), number of fruits per plant (NRF), estimated yield per plant (YIE), total soluble solids (°BRIX), seed weight (SW), number of seeds per fruit (SxF), and fruit shape index (FRUSI). Accessions are marked according to the genotype at the *Fasciated* locus, being wild-type (WT, red) or *fas* mutant (fas, light blu). Ellipses represent 68% confidence interval for each group.

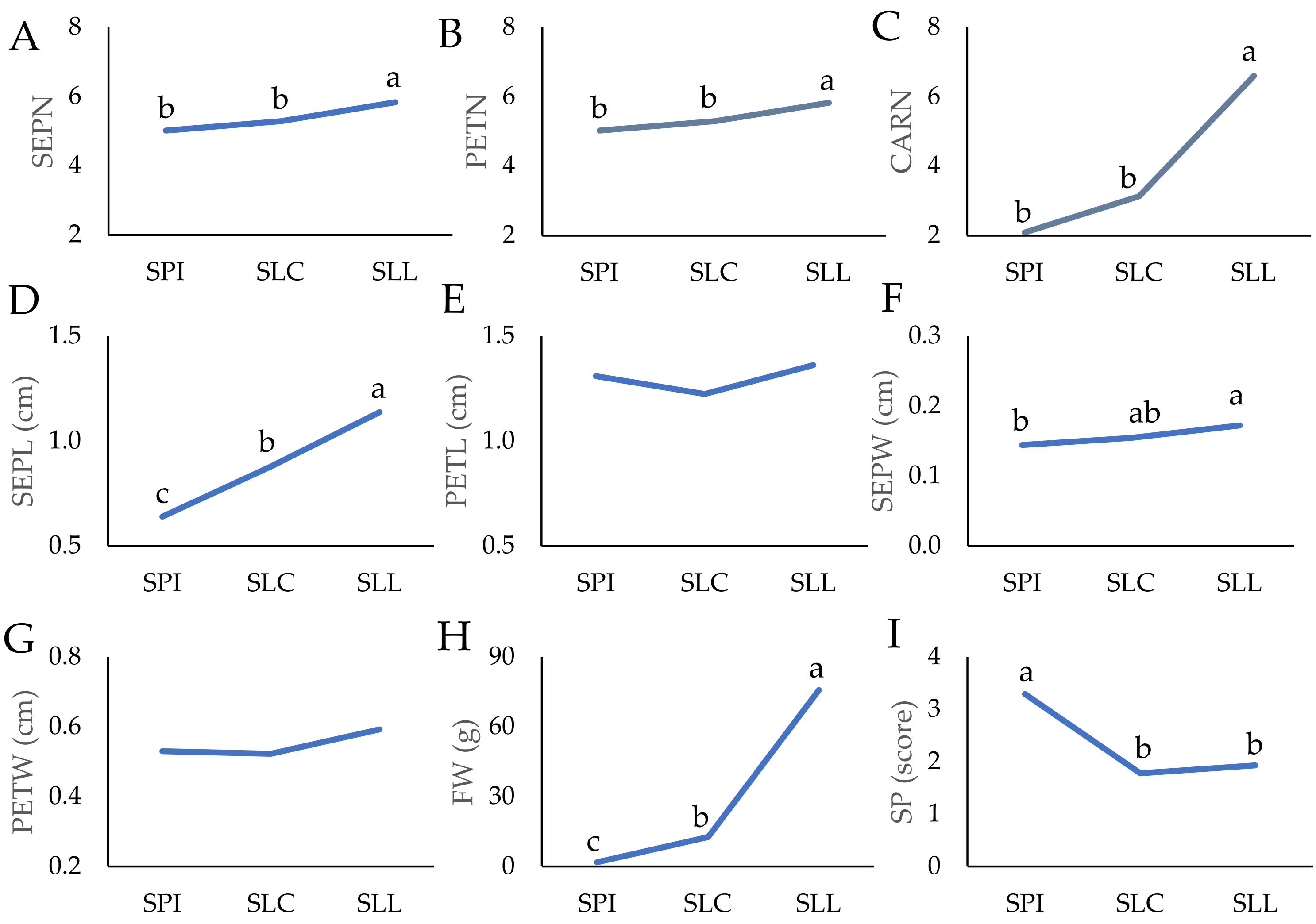

Supplementary Figure S5. Analysis of variance of floral and reproduction-related data from [16] as distributed in *Solanum pimpinellifolium* (SPI*), S. lycopersicum* var. *cerasiforme* (SLC), and *S. lycopersicum* var. *lycopersicum* (SLL). Traits reported are (**A**) sepal (SEPN), (**B**) petal (PETN) and (**C**) carpel (CARN) number; (**D**) sepal (SEPL) and (**E**) petal (PETL) length; (**F**) sepal (SEPW) and (**G**) petal (PETW) width, (**H**) fruit weight (FW), and (**I**) stigma position (SP). For each series, means indicated by the same lowercase letter are not significantly different after Duncan's mean separation test ($p \leq 0.05$).
